# Conflicts of interest in research on electronic cigarettes

**DOI:** 10.18332/tid/90668

**Published:** 2018-06-01

**Authors:** Cristina Martínez, Marcela Fu, Iñaki Galán, Mónica Pérez-Rios, Jose M. Martínez-Sánchez, Maria J. López, Xisca Sureda, Agustín Montes, Esteve Fernández

**Affiliations:** 1 Tobacco Control Unit, Cancer Control and Prevention Programme, Institut Català d’Oncologia-ICO, Barcelona, Spain; 2 Cancer Control and Prevention Group, Institut d’Investigació Biomèdica de Bellvitge-IDIBELL, Barcelona, Spain; 3 Department of Nursing, Faculty of Medicine and Health Sciences, Universitat Internacional de Catalunya, Barcelona, Spain; 4 Department of Public Health, Mental Health and Perinatal Nursing, Faculty of Medicine and Health Sciences, Universitat de Barcelona, Barcelona, Spain; 5 Department of Clinical Sciences, Faculty of Medicine and Health Sciences, Universitat de Barcelona, Barcelona, Spain; 6 National Centre for Epidemiology, Instituto de Salud Carlos III, Madrid, Spain; 7 Department of Preventive Medicine and Public Health, School of Medicine, Universidad Autónoma de Madrid/IdiPAZ, Madrid, Spain; 8 Epidemiology Unit, Galician Directorate for Public Health, Galician Health Authority, Santiago de Compostela, Spain; 9 Department of Preventive Medicine and Public Health, School of Medicine, Universidade de Santiago de Compostela, Santiago de Compostela, Spain; 10 Consortium for Biomedical Research in Epidemiology & Public Health (CIBERESP), Madrid, Spain; 11 Group of Evaluation of Health Determinants and Health Policies, Department of Medicine, Faculty of Medicine and Health Sciences, Universitat Internacional de Catalunya, Barcelona, Spain; 12 Evaluation and Intervention Methods Service, Agència de Salut Pública de Barcelona, Barcelona, Spain; 13 Sant Pau Institute of Biomedical Research (IIB Sant Pau), Barcelona, Spain; 14 Social and Cardiovascular Epidemiology Research Group, School of Medicine, Universidad de Alcalá, Madrid, Spain

**Keywords:** conflicts of interest, electronic cigarettes, bibliography as topic

## Abstract

**INTRODUCTION:**

The tobacco control community has raised some concerns about whether studies on electronic cigarettes (e-cigarettes) published in scientific journals hide conflicts of interest (COI) and whether such reports are biased. This study assessed potential COI in the e-cigarette scientific literature.

**METHODS:**

Cross-sectional study was conducted on e-cigarette publications indexed in PubMed up to August 2014. We extracted information about the authors (affiliations, location, etc.), publication characteristics (type, topic, subject, etc.), results and conclusions, presence of a COI statement, and funding by and/or financial ties to pharmaceutical, tobacco, and/or e-cigarette companies. An algorithm to determine the COI disclosure status was created based on the information in the publication. Prevalence ratios (PRs) and confidence intervals (CIs) were calculated to identify associations with COI disclosure, controlling for several independent variables.

**RESULTS:**

Of the 404 publications included in the analysis, 37.1% (n=150) had no COI disclosure statement, 38.6% declared no COI, 13.4% declared potential COI with pharmaceutical companies, 3.0% with tobacco companies, and 10.6% with e-cigarette companies. The conclusions in publications with COI, which were mainly tied to pharmaceutical companies, were more likely to be favourable to e-cigarette use (PR=2.23; 95% CI: 1.43–3.46). Publications that supported the use of e-cigarettes for both harm reduction (PR=1.81; 95%CI: 1.14–2.89) and smoking cessation (PR=2.02; 95% CI: 1.26–3.23) were more likely to have conclusions that were favourable to e-cigarettes.

**CONCLUSIONS:**

One-third of the publications reporting studies on e-cigarettes did not have a COI disclosure statement, and this proportion was even higher in news articles, editorials and other types of publications. Papers with conclusions that were favourable to e-cigarette use were more likely to have COI. Journal editors and reviewers should consider evaluating publications, including funding sources, to determine whether the results and conclusions may be biased.

## INTRODUCTION

A conflicts of interest (COI) is ‘a set of conditions in which professional judgment concerning a primary interest (such as a patient’s welfare or the validity of research) tends to be unduly influenced by a secondary interest (such as financial gain)’^1^. COI occurs when authors, reviewers or editors have interests that are not fully apparent and that could influence their judgment on what they publish or review^2,3^.

Efforts to provide transparency regarding COIs date back to 1984^4,5^. In recent years, COI disclosure policies have become a regular part of biomedical research. The International Committee of Medical Journal Editors’ guidelines recommend that authors should disclose the study’s funding sources and any financial ties to core companies, such as pharmaceutical or tobacco companies^6^. In addition, since 2013 the Committee on Publication Ethics (COPE) established two levels of obligation for its members. The first is a Code of Conduct which Journal Editors are expected to practise (and will consider complaints against members who do not follow it), and the second is a set of Best Practice recommendations, which are voluntary, but are suggested to include into the journal’s policies and practices^7^. Among these practices, ‘Editors should have systems for managing COI by authors and reviewers, as well as their own COI, including their staff and editorial board members’^7^.

Nevertheless, evidence shows that tobacco research sponsored by pharmaceutical^4^ or tobacco companies may be biased^8-11^ due to vested economic interests. In the 1970s, tobacco companies downplayed the harm of second-hand smoke, for example, by hiding damaging data and distorting research findings^9-12^. Nowadays, the four major tobacco companies are involved in the manufacturing of electronic cigarettes (also called e-cigarettes). Considering that this electronic device resembles cigarette use, it is plausible that the same strategies could be used for manipulating the scientific evidence about the use of this relatively new product^12^. In fact, some in the scientific community are concerned that publications on e-cigarettes may hide COI and thus their results could be biased^13-16^. A systematic review of the health effects of e-cigarettes revealed that 34% of the 76 reviewed articles had declared COI, mostly funding from the manufacturers of e-cigarettes or authors who had acted as consultants for the manufacturers of smoking cessation treatments^17^. Another systematic review that addressed passive exposure to e-cigarettes showed that 30% of the 40 reviewed papers had potential COI^18^. One report on the ‘evidence update’ of e-cigarettes^19^ claimed that ‘e-cigarettes are 95% less harmful to health than normal cigarettes’; this claim was contested because its conclusions appeared to be biased due to COI^12,15^. That report also raised serious questions because of its lack of a peer-review process, and thus several organizations put this issue in the spotlight^12,19^.

Some journals do not accept manuscripts with links to tobacco companies^20^. This decision has been criticised by some authors, who argue that these journals have passed a strong rule without enough evidence to justify it and that the journals themselves have some countervailing reasons to do so^20^. Although full disclosure of financial ties is common in scientific journals when publishing an original report, it seems to be less a priority for other types of articles, such as commentaries, news items, etc^7^. There are hundreds of publications about e-cigarettes, but, considering its novelty, less than half are original articles or reviews, with the rest being mainly notes, editorials or opinions^21^.

Thus, our objective was to conduct an assessment of potential COI in the emergent scientific literature on e-cigarettes. Specifically, we aimed to analyse the declaration of potential COI in any type of publication (original and non-original reports), according to the criteria proposed by COPE.

## METHODS

We performed a search on PubMed (www.pubmed. gov) to identify all publications on e-cigarettes listed in Medline and indexed until August 2014. We included all types of publications whose primary or secondary aim or topic was related to e-cigarettes.

The full search strategy used was as follows: (‘electronic cigarettes’[All Fields] OR e cig[All Fields] OR e cigar[All Fields] OR e cigarettes[All Fields] OR e cigarette[All Fields] OR e cigarette’s[All Fields] OR e cigs[All Fields] OR ‘electronic nicotine delivery system’[All Fields] OR ‘electronic nicotine delivery devices’[All Fields]) AND (‘0001/01/01’[PDAT]: ‘2014/08/31’[PDAT]). No other restrictions were established.

**Figure 1:**
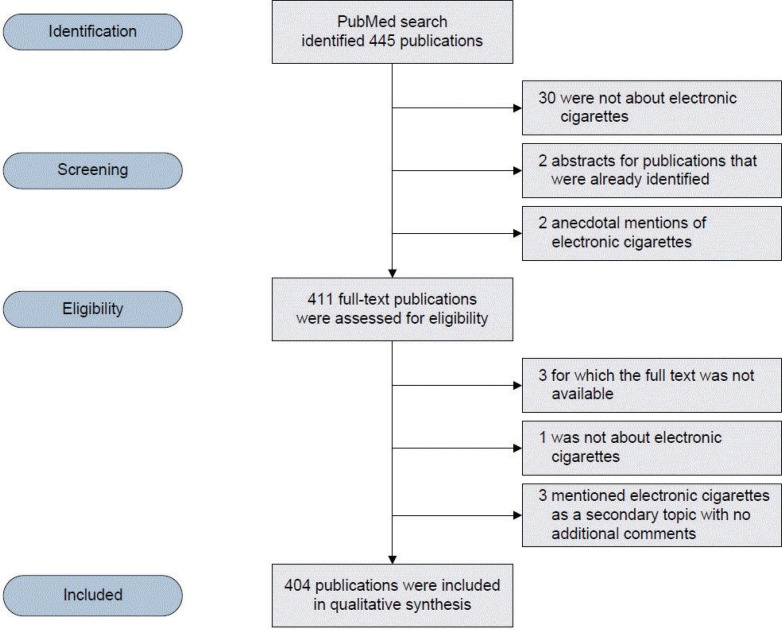
PRISMA chart showing the selection of the publications included in the analysis.

The search returned 445 publications. Five pairs of reviewers assessed the title and abstracts (if available) of about 90 publications each. Of the retrieved publications, 30 were not related to e-cigarettes, 2 were duplicate publications, and 2 anecdotally mentioned e-cigarettes. Thus, 411 publications were eligible for full assessment. After reading the full text, 7 were excluded: 1 was not related to electronic cigarettes, 3 mentioned them as a secondary topic with no specific comments, and 3 were unavailable to retrieve (including unsuccessful contact with the corresponding authors); thus, 404 publications were included in the final analysis (Figure 1).

Some tools were created to extract the information, including a protocol to guide the pull out of data, an algorithm (Supplementary Figure S1) to assess the publication’s disclosure status, and an *ad hoc* Excel spreadsheet to register the information for each publication. They were pilot-tested using 5 full-text publications that were assessed by all of the researchers. When there were any questions, the reviewers discussed the papers with the assigned partner until agreement was reached. If they could not come to an agreement, two other researchers were assigned and resolved the issue.

### Variable definitions and operationalisation

Based on information in the publication several variables were assessed, including information about the authors, the publications, COI, results and conclusions.

#### Authors’ information

Authors: The surname and initials for all authors were collected.

Institutional affiliation: All affiliations from all the authors were considered. This information was classified as follows: no affiliations were provided for any of the authors; some of the authors had their affiliations specified; all authors had their affiliations specified. Affiliations were classified into the following categories: 1) academy/university, 2) governmental/public agency, 3) health organization, 4) private company, 5) pharmaceutical company, 6) tobacco company, 7) e-cigarette company, and 8) other. If the affiliation was related to more than one core company (pharmaceutical, tobacco, or e-cigarette companies, PTEC_CO), then it was classified based on its main scope (for instance, in the case of a tobacco company that also manufactures e-cigarettes, it was classified as ‘tobacco company’).

Country of origin: The country with which the first author was affiliated was recorded and was reclassified according to continent for the analysis.

#### Publication characteristics

Year of publication: The search included all publications indexed in PubMed up to August 2014 (publications that were in press when the search was conducted were updated to reflect the final date of publication).

Type of publication: Publications were classified as original article, review, editorial, letter, news, commentary, or other, based on the information in the full publication; when this information was not provided, the classification was based on the structure of the publication, following the descriptions of different types of publications on the journal’s website.

Aim: The presence of a specific research aim was captured (no, yes).

Empirical data: The reporting of empirical data was determined (no, yes). For publications reporting empirical data, they were classified as follows: 1) the main topic/s of the study-allowing for multiple responses such as prevalence of use, toxicology (chemical analysis), health effects, smoking cessation, topography, attitudes and knowledge, regulation and marketing, clinical advice, other, and miscellaneous (different or mixed topics), and 2) the main subject of the study-allowing for multiple responses such as no main subject, humans, e-cigarettes themselves, including the device, liquids and emissions, regulation and marketing, and other.

#### Conflicts of interest (COI)

COI: The presence of a COI section was determined (no, yes), using this or similar wording (e.g. competing interests). In addition, we recorded whether the authors reported their COI status in any section of the publication (no, yes) and the literal wording. Also, we recorded whether the potential COI reported was related to pharmaceutical (P), tobacco (T) and/or electronic cigarette (EC) companies (PTEC_CO) (no, yes).

Acknowledgments: The presence of an acknowledgments section was assessed (no, yes). If yes, we recorded whether the authors acknowledged any of the PTEC_CO.

Funding: The disclosure of any funding or sponsorship for the study (grant, donation, other) in any section of the publication was assessed (no, yes). If yes, we classified the source(s) of funding as being from a university, governmental/public organisation, private organisation, PTEC_CO, and/or other sources.

Financial ties: We determined whether the authors had other financial ties to PTEC_CO (no, yes) and the type of these financial ties, meaning personal payments for: 1) services, travel, training, etc.; 2) money for patents, conferences, etc.; and 3) other financial ties. This information was drawn from any part of the publication, main affiliation, COI statement, funding and acknowledgements.

Overall view of disclosure status: Based on the information about the funding sources and the authors’ financial ties, we classified the disclosures of potential COI into 5 categories as follows: the authors did not disclose any COI within the manuscript (code 0); the authors disclosed no COI for the study, and no funding from any PTEC_CO are mentioned (code 1); the authors disclosed no COI for the study, but they acknowledged that some of the authors or previous studies conducted by the authors received funding from PTEC_CO in the past (code 2); the authors disclosed no COI for the study, but acknowledged receiving funding for the study from PTEC_CO (code 3); and the authors disclosed COI with PTEC_CO (code 4). For multivariate Poisson regression analysis, we excluded publications with no disclosures (code 0) and collapsed the publications into two categories: those that disclosed no COI at all (code 1) and those that disclosed any COI (codes 2–4).

#### Results, conclusions and publication tendency

Results: For empirical publications, the reported results were categorised as being ‘favourable’ to the use of e-cigarettes, ‘not being favourable to their use’, ‘neutral’ (not in favour nor against), or ‘unclear’ (undefined tendency). The classification was done according to the findings provided in the study, avoiding any interpretation of them. For the analysis, we combined publications in the ‘neutral’ and ‘unclear’ categories into one category.

Conclusions: For all publications, with and without results, the specific conclusions in this section, or in the abstract if unclear, were also categorised as being ‘favourable’ to the use of e-cigarettes, ‘not being favourable’ to their use, ‘neutral’ or ‘unclear’. For assessing this variable we searched for authors’ conclusions and recommendations in the corresponding section, in the discussion section or in the abstract if it was not clear, in the case of empirical publications, and within the publication for non-empirical publications. We also combined the ‘neutral’ and ‘unclear’ conclusions into one category.

Overall publication tendency: Based on the results and conclusions, we determined whether the publication showed a tendency to support some of the following (multiple responses were possible): the use of e-cigarettes for harm reduction (reduction of tobacco products by using e-cigarettes), stronger regulation of e-cigarettes, and the use of e-cigarettes for tobacco smoking cessation.

### Statistical analysis

We report the frequency and percentage of COI disclosure (codes 0–4), the potential COI with PTEC_CO and the tendency of the main conclusions of publications with empirical data according to the publication characteristics, the main study subjects and the topics studied. We also describe the COI disclosure (codes 0–4) according to the conclusion and the publication tendency, for all publications and for empirical studies. In addition, we calculated prevalence ratios (PRs) and their 95% confidence intervals (CIs) using multivariate Poisson regression analysis with robust standard error estimations (White-Huber robust standard errors) to identify associations with COI disclosure, controlling for the continent of the first author, the year of publication, empirical data, main subject and main topic.

## RESULTS

A total of 404 publications were analysed. The more recent the publication year, the higher the number of publications on e-cigarettes, with a tendency towards an exponential increase (Supplementary Figure S2). Of all publications, 36.9% were original articles (Table 1). Almost all (96.0%) were written in English; the first author was in North America in half of the publications (48.5% of all publications were from the USA and 2.2% from Canada), and in Europe in a third of them (11.9% of all publications were from UK, 6.4% from Italy and 3.5% from Greece). Information about the country could not be determined in 7.7% of all the publications. The most frequent topics were e-cigarette regulation and marketing (24.5%) and toxicology (13.1%); nevertheless, a high percentage of the publications focused on miscellaneous topics, i.e. covering a variety of topics in the same publication or not addressing specific topics (21.5%). Overall, 41.1% of the publications reported empirical data; in these publications, the most frequent study subject was humans (62.0%).

**Table 1:** Disclosure of conflicts of interest and potential conflicts of interest with core companies (PTEC_COs^a^)

	*Total*	*Conflicts of interest disclosure status*					*Potential conflicts of interest with core companies^b^*		

		*No disclosure (code 0 )*	*Disclosed no COIs (code 1 )*	*Disclosed COIs for previous studies or for some authors (code 2 )*	*Disclosed no COIs, but acknowledge financial support by PTEC_COs^a^ (code 3 )*	*Disclosed potential COIs (code 4 )*	*Pharmaceutical companies*	*Tobacco companies*	*E-cigarette companies*

	*n*	*n (%)*	*n (%)*	*n (%)*	*n (%)*	*n (%)*	*n (%)*	*n (%)*	*n (%)*
**All publications**	404	150 (37.1)	156 (38.6)	25 (6.2)	18 (4.5)	55 (13.6)	54 (13.4)	12 (3.0)	43 (10.6)
**Type of publication**									
Original article	149	34 (22.8)	65 (43.6)	13 (8.7)	12 (8.1)	25 (16.8)	24 (16.1)	4 (2.7)	23 (15.4)
Review	44	11 (25.0)	18 (40.9)	4 (9.1)	2 (4.5)	9 (20.5)	10 (22.7)	4 (9.1)	4 (9.1)
Editorial	25	16 (64.0)	8 (32.0)	0 (0.0)	0 (0.0)	1 (4.0)	0 (0.0)	0 (0.0)	0 (0.0)
Letter	35	7 (20.0)	19 (54.3)	1 (2.9)	1 (2.9)	7 (20.0)	5 (14.3)	1 (2.9)	8 (22.9)
News	42	42 (100.0)	0 (0.0)	0 (0.0)	0 (0.0)	0 (0.0)	0 (0.0)	0 (0.0)	0 (0.0)
Commentary	20	2 (10.0)	10 (50.0)	4 (20.0)	1 (5.0)	3 (15.0)	6 (30.0)	1 (5.0)	3 (15.0)
Other	89	38 (42.7)	36 (40.4)	3 (3.4)	2 (2.2)	10 (11.2)	9 (10.1)	2 (2.2)	5 (5.6)
**Language^c^**									
English	388	140 (36.1)	152 (39.2)	24 (6.2)	18 (4.6)	54 (13.9)	52 (13.4)	12 (13.1)	42 (10.8)
Other	16	10 (62.5)	4 (25.0)	1 (6.3)	0 (0.0)	1 (6.3)	2 (12.5)	0 (0.0)	1 (6.3)
**Continent of first author**									
North America^d^	205	61 (29.8)	107 (52.2)	4 (2.0)	14 (6.8)	19 (9.3)	14 (6.8)	4 (2.0)	5 (2.4)
Africa	4	3 (75.0)	1 (25.0)	0 (0.0)	0 (0.0)	0 (0.0)	0 (0.0)	0 (0.0)	1 (25.0)
Asia	12	8 (66.7)	4 (33.3)	0 (0.0)	0 (0.0)	0 (0.0)	0 (0.0)	0 (0.0)	0 (0.0)
Europe	137	46 (33.6)	36 (26.3)	20 (14.6)	3 (2.2)	32 (23.4)	36 (26.3)	7 (5.1)	32 (23.4)
Oceania	15	3 (20.0)	6 (40.0)	1 (6.7)	1 (6.7)	4 (26.7)	4 (26.7)	1 (6.7)	5 (33.3)
Unknown	31	29 (93.5)	2 (6.5)	0 (0.0)	0 (0.0)	0 (0.0)	0 (0.0)	0 (0.0)	0 (0)
**Main topic**									
Prevalence	43	14 (32.6)	16 (37.2)	2 (4.7)	4 (9.3)	7 (16.3)	5 (11.6)	1 (2.3)	3 (7.0)
Toxicology	53	22 (41.5)	12 (22.6)	4 (7.5)	4 (7.5)	11 (20.8)	7 (13.2)	1 (1.9)	10 (18.9)
Health effects	39	9 (23.1)	19 (48.7)	3 (7.7)	2 (5.1)	6 (15.4)	5 (12.8)	2 (5.1)	9 (23.1)
Smoking cessation	44	10 (22.7)	16 (36.4)	5 (11.4)	1 (2.3)	12 (27.3)	16 (36.4)	2 (4.5)	10 (22.7)
Topography	3	0 (0.0)	3 (100.0)	0 (0.0)	0 (0.0)	0 (0.0)	0 (0.0)	0 (0.0)	0 (0.0)
Attitudes, knowledge	22	7 (31.8)	10 (45.5)	2 (9.1)	1 (4.5)	2 (9.1)	3 (13.6)	1 (4.5)	0 (0.0)
Regulation, marketing	99	46 (46.5)	44 (44.4)	3 (3.0)	2 (2.0)	4 (4.0)	3 (3.0)	0 (0.0)	4 (4.0)
Clinical advice	3	1 (33.3)	1 (33.3)	0 (0.0)	0 (0.0)	1 (33.3)	1 (33.3)	0 (0.0)	0 (0.0)
Other	11	3 (27.3)	6 (54.5)	0 (0.0)	1 (9.1)	1 (9.1)	0 (0.0)	1 (9.1)	0 (0.0)
Miscellaneous	87	38 (43.7)	29 (33.3)	6 (6.9)	3 (3.4)	11 (12.6)	14 (16.1)	4 (4.6)	7 (8.0)
**Empirical data**									
Yes	166	37 (22.3)	77 (46.4)	13 (7.8)	12 (7.2)	27 (16.3)	25 (15.1)	5 (3.0)	25 (15.1)
No	238	113 (47.5)	79 (33.2)	12 (5.0)	6 (2.5)	28 (11.8)	29 (12.2)	7 (2.9)	18 (7.6)
**Main subject^e^ (n=166)**									
Humans	103	20 (19.4)	46 (44.7)	7 (6.8)	9 (8.7)	21 (20.4)	20 (19.4)	4 (3.9)	19 (18.4)
E-cigarettes per se	37	12 (32.4)	15 (40.5)	3 (8.1)	2 (5.4)	5 (13.5)	4 (10.8)	1 (2.7)	5 (13.5)
Regulation, marketing	19	3 (15.8)	13 (68.4)	2 (10.5)	0 (0.0)	1 (5.3)	1 (5.3)	0 (0.0)	1 (5.3)
Other^f^	7	2 (28.6)	3 (42.9)	1 (14.3)	1 (14.3)	0 (0.0)	0 (0.0)	0 (0.0)	0 (0.0)

a PTEC_COs: pharmaceutical (P), tobacco (T) and/or electronic cigarette (EC) companies.

b Potential conflicts of interest with core companies are not exclusive.

c There was 1 publication in both English and French that was codified as an English language publication.

d All publications were from the United States or Canada.

e Publications with empirical data only.

f Other subjects: in vitro studies, animal studies, or other.

Table 1 shows the COI disclosures in the publications. Of the included publications, 150 (37.1%) did not include any disclosure related to COI, mainly those without empirical data, including all 42 of the news articles and 16 of the 25 editorials. Another 38.6% of the publications disclosed no COI at all; 6.2% stated that there were no COI, but some authors had potential COI in previous publications; while 4.5% stated that they had no COI, but they acknowledged financial support by PTEC_CO. Finally, 13.6% of the publications disclosed potential COI: 13.4% with pharmaceutical companies, 3.0% with tobacco companies, and 10.6% with e-cigarette companies.

Table 2 shows the tendencies of the conclusions regarding e-cigarettes for the publications that had empirical data, both overall and according to independent variables (n=164; 2 publications had no conclusions). Of these, 55.5% of the publications had conclusions that were neutral or unclear, and 22.6% had favourable conclusions about e-cigarettes. Most of the publications were original articles, so the conclusion tendencies were similar to those observed in the publications overall. Publications in which the main topic was related to smoking cessation were the most prevalent in terms of having conclusions favourable to the use of e-cigarettes.

**Table 2:** The tendencies of the main conclusions of publications on electronic cigarettes that reported empirical data

		*Conclusion*		

	*Total*	*Favourable n (%)*	*Not favourable n (%)*	*Neutral/unclear n (%)*
**All publications^a^**	164	37 (22.6)	36 (22.0)	91 (55.5)
**Type of publication**				
Original article	142	36 (25.4)	28 (19.7)	78 (54.9)
Review	9	0 (0.0)	0 (0.0)	9 (100.0)
Letter	5	0 (0.0)	4 (80.0)	1 (20.0)
Editorial, news, commentary	0	0 (0.0)	0 (0.0)	0 (0.0)
Other	8	1 (12.5)	4 (50.0)	3 (37.5)
**Language^b^**				
English	161	36 (22.4)	35 (21.7)	90 (55.9)
Other	3	1 (33.3)	1 (33.3)	1 (33.3)
**Continent of first author**				
North America^c^	104	11 (10.6)	29 (27.9)	64 (61.5)
				
Africa	1	1 (100.0)	0 (0.0)	0 (0.0)
Asia	7	0 (0.0)	3 (42.9)	4 (57.1)
Europe	49	23 (46.9)	4 (8.2)	22 (44.9)
Oceania	3	2 (66.7)	0 (0.0)	1 (33.3)
**Main subject**				
Humans	102	31 (30.4)	19 (18.6)	52 (51.0)
E-cigarettes per se	36	4 (11.1)	10 (27.8)	22 (61.1)
Regulation, marketing	19	0 (0.0)	6 (31.6)	13 (68.4)
Other^d^	7	2 (28.6)	1 (14.3)	4 (57.1)
**Main topic**				
Prevalence	36	5 (13.9)	9 (25.0)	22 (61.1)
Toxicology	34	5 (14.7)	7 (20.6)	22 (64.7)
Health effects	20	8 (40.0)	2 (10.0)	10 (50.0)
Smoking cessation	16	13 (81.3)	1 (6.3)	2 (12.5)
Topography	3	1 (33.3)	1 (33.3)	1 (33.3)
Attitudes, knowledge	21	2 (9.5)	4 (19.0)	15 (71.4)
Marketing, regulation	25	0 (0.0)	10 (40.0)	15 (60.0)
Clinical advice	0	0 (0.0)	0 (0.0)	0 (0.0)
Other	7	3 (42.9)	1 (14.3)	3 (42.9)
Miscellaneous	2	0 (0.0)	1 (50.0)	1 (50.0)

a Two publications had no conclusions and thus they were excluded.

b There was 1 publication in both English and French that was codified as an English language publication.

c All publications were from the United States or Canada.

d Other subjects: cells, animals, miscellaneous subjects.

Table 3 shows the distribution of the types of COI disclosures among all publications and among those with empirical data, according to the conclusion and the publication tendencies. Overall publications with conclusions that tended to favour the use of e-cigarettes, 36.7% disclosed having potential COI, whereas 40.8% of those with conclusions that tended to be neutral/unclear disclosed no COI. When we consider only the 166 empirical studies, 37.8% of those with conclusions that were favourable to the use of e-cigarettes disclosed COI, whereas the majority of those with conclusions that tended not to favour the use of e-cigarettes or that were neutral/unclear disclosed that they did not have COI (55.6% and 50.5%, respectively). Regarding the distribution of COI disclosures according to the publication tendencies, we observed that 20.5% of the overall publications supported the use of e-cigarettes as a harm reduction tool, 31.7% supported regulating e-cigarettes, and 17.6% supported their use for smoking cessation. Among the empirical studies, most that supported harm reduction or e-cigarette regulation disclosed no COI (40.0% and 52.3%, respectively), whereas those publications that supported smoking cessation mostly disclosed potential COI.

**Table 3:** Disclosure of conflicts of interest (COIs) according to the publication’s conclusions and the tendency to be favourable or not favourable to the use of e-cigarettes

	*Total*	*No disclosure (code 0 )*	*Disclosed no COIs (code 1 )*	*Disclosed COIs for previous studies or for some authors (code 2 )*	*Disclosed no COIs, but acknowledge financial support by PTEC_COs^a^ (code 3 )*	*Disclosed potential COIs (code 4 )*

	*n*	*n (%)*	*n (%)*	*n (%)*	*n (%)*	*n (%)*
**All publications**	404	150 (37.1)	156 (38.6)	25 (6.2)	18 (4.5)	55 (13.6)
**Conclusion**						
Favourable	79	11 (13.9)	23 (29.1)	14 (17.7)	2 (2.5)	29 (36.7)
Not favourable	93	33 (35.5)	53 (57.0)	0 (0.0)	2 (2.2)	5 (5.4)
Neutral/unclear	169	64 (37.9)	69 (40.8)	8 (4.7)	14 (8.3)	14 (8.3)
No conclusion	63	42 (66.7)	11 (17.5)	3 (4.8)	0 (0.0)	7 (11.1)
**Publication tendency^b^**						
Supports their use for harm reduction	83	24 (28.9)	23 (27.7)	11 (13.3)	2 (2.4)	23 (27.7)
Supports regulation	128	55 (43.0)	51 (39.8)	9 (7.0)	4 (3.1)	9 (7.0)
Supports their use for smoking cessation	71	18 (25.4)	18 (25.4)	10 (14.1)	2 (2.8)	23 (32.4)
**Empirical studies**	166	37 (22.3)	77 (46.4)	13 (7.8)	12 (7.2)	27 (16.3)
**Conclusion**						
In favour	37	2 (5.4)	11 (29.7)	8 (21.6)	2 (5.4)	14 (37.8)
Not in favour	36	14 (38.9)	20 (55.6)	0 (0.0)	1 (2.8)	1 (2.8)
Neutral/unclear	91	20 (22.0)	46 (50.5)	5 (5.5)	9 (9.9)	11 (12.1)
No conclusion	2	1 (50.0)	0 (0.0)	0 (0.0)	0 (0.0)	1 (50.0)
**Publication tendency^b^**						
Supports their use for harm reduction	25	3 (12.0)	10 (40.0)	4 (16.0)	2 (8.0)	6 (24.0)
Supports regulation	44	13 (29.5)	23 (52.3)	5 (11.4)	2 (4.5)	1 (2.3)
Supports their use for smoking cessation	30	3 (10.0)	8 (26.7)	6 (20.0)	2 (6.7)	11 (36.6)

a PTEC_COs: pharmaceutical (P), tobacco (T) and/or electronic cigarette (EC) companies.

b Multiple responses were possible.

Table 4 shows the PRs of the publications with favourable conclusions about e-cigarettes and the publications’ tendencies, estimated from multivariate Poisson regression models, according to having a COI disclosure, the main topic of research, and the main subject studied. Publications with a COI disclosure were more likely to have favourable conclusions about e-cigarettes (PR=2.23; 95% CI: 1.43–3.46), to support its use as a tool for harm reduction (PR=1.81; 95% CI: 1.14–2.89), and to support its use for smoking cessation (PR=2.02; 95% CI: 1.26–3.23). Publications about e-cigarette toxicology/health effects or about smoking cessation/clinical advice were more likely to have conclusions that favoured the use of e-cigarettes. Furthermore, papers in which the main subject was e-cigarettes themselves were less likely to have conclusions that were favourable to e-cigarettes (PR=0.49; 95% CI: 0.29–0.85). Publications with COI related to pharmaceutical companies were more likely to have favourable conclusions about e-cigarettes (PR=1.59; 95% CI: 1.05–2.40); this association was not found for COI involving other types of companies (data not shown). Publications that addressed smoking cessation/clinical advice, and other or miscellaneous topics, were more likely to support smoking cessation (PR=3.10 and 2.71, respectively), whereas papers in which the main subject was e-cigarettes themselves were less likely to support the use of e-cigarettes for smoking cessation (PR=0.30; 95% CI: 0.15–0.60).

**Table 4:** Conclusions and publications’ tendencies that favour e-cigarettes use according to conflicts of interest disclosure, the main research topic, and the main subject of the study

			*Publications’ tendency*					

	*Conclusion that favour use of e-cigarettes*		*Supports their use for harm reduction*		*Supports regulation*		*Supports their use for smoking cessation*	

*Variables*	*PR*	*95% CI*	*PR*	*95% CI*	*PR*	*95% CI*	*PR*	*95% CI*
Conflicts of interest disclosure (yes^a^)	2.23	1.43–3.46	1.81	1.14–2.89	0.78	0.49–1.24	2.02	1.26–3.23
**Main topic**								
Prevalence/topography/attitudes	Ref.							
Toxicology/health effects	2.04	1.03–4.05	1.82	0.67–4.93	0.70	0.30–1.63	1.20	0.53–2.71
Smoking cessation/clinical advice	3.01	1.55–5.85	2.58	0.96–6.91	0.48	0.16–1.42	3.10	1.53–6.28
Regulation/marketing	2.08	0.91–4.78	2.97	1.05–8.44	1.31	0.55–3.11	2.48	0.90–6.85
Other/miscellaneous	1.97	0.86–4.51	2.34	0.82–6.73	1.27	0.49–3.28	2.71	1.13–6.50
**Main subject**								
No subject	Ref.							
Humans	0.89	0.43–1.87	0.94	0.44–2.00	0.54	0.23–1.26	0.85	0.36–2.00
E-cigarettes per se	0.49	0.29–0.85	0.58	0.34–0.98	0.88	0.50–1.53	0.30	0.15–0.60
Regulation, marketing	0.38	0.11–1.23	0.34	0.11–1.00	0.89	0.42–1.87	NA^b^	NA^b^
Other	0.60	0.22–1.68	0.83	0.24–2.82	0.42	0.07–2.71	0.25	0.04–1.36

PR: Prevalence ratios were estimated by Poisson regression adjusted for main topic, main subject, empirical data, continent of the first author, and publication year.

a Yes: This includes codes 2–4 (Supplementary Figure S1).

b NA: Not applicable (i.e. the values were very unstable due to limited data).

## DISCUSSION

We examined the factors associated with reporting potential COI in the scientific literature on e-cigarettes. We found that over a third of the publications did not have any COI disclosures; these were mostly news, editorials and other types of publications. Authors who had COI (disclosed or not) and publications in which the main topics included toxicology/health effects or smoking cessation/clinical advice were more likely to report favourable conclusions about e-cigarettes. In contrast, studies in which the main subject was e-cigarettes themselves were less likely to have favourable conclusions about e-cigarettes. There are a few possible explanations for these findings. First, the authors who published studies with favourable conclusions about e-cigarettes could have some ties to PTEC_CO. These authors may have framed the research question, conducted the study, or reported the results and conclusions, either consciously or unconsciously with the interests of their sponsors in mind. Second, it is possible that the authors failed to disclose their sponsorship or research constraints, and the editors were not as strict as with studies that reported non-favourable conclusions.

We found that the conclusions of reports on e-cigarettes are associated with their authors’ COI. In addition, we observed that publications that supported the use of e-cigarettes for smoking reduction and smoking cessation were more likely to be from authors with COI with PTEC_CO. Our study shows that publications that report favourable conclusions about e-cigarettes come from authors who had received financial support from PTEC_CO. This pattern is seen in publications with both empirical and non-empirical data.

A systematic review of the prevalence of COI found that a third of biomedical researchers in academic institutions had COI^1^. Studies measuring undisclosed COI suggest that between 43% and 69% of study reports and other publications fail to include disclosures of COI^3^. In our study, we found that 37.1% of publications did not include any disclosure about COI. Disclosure only reveals the possibility of bias, without providing any guidance for resolving it^3^. Biases introduced by COI are difficult to deal with and should be treated like any other confounder that could affect the results of a study^3^; however, treating COI as a confounder is challenging. The failure of the scientific community to effectively address COI has serious repercussions for public health policy^7^. The scientific community must provide transparency, supervision and accountability to help ensure that research is not biased by undisclosed COI^7^. Some suggested methods for improving the disclosure of potential COI include the introduction of clinical trial registries, public accessible registers for declarations of interest, and the creation of an Ethics Centre that monitors and reports the enforcement of ethical standards^7^. A compulsory detailed statement of COI, including some of the variables studied in this analysis, may disclose them in a more effective manner. This would not completely mitigate the risk of bias, but it would facilitate a better understanding of how COI are related to biases and how they can influence research consensus^3^.

We also found that the type of publication can influence its tendency to e-cigarettes. Practically all reviews were neutral in terms of the tone of their conclusions, but letters and other non-empirical publications were more likely to be either against or favourable to the regulation of e-cigarettes. The journal characteristics may also influence the results and conclusions of the studies, as the quality of their reporting may vary according to the journal’s standards^22^. Articles published in peer-reviewed journals are generally of higher quality than those published in non-peer-reviewed journals^2^. However, we did not include this independent variable in our assessment. Additional research is needed to clarify possible associations between the journal’s quality and the disclosure of COI in e-cigarette publications.

There is evidence that financial COI lead to systematic bias^23,24^. In our study, we observed that only publications in which the authors reported ties with pharmaceutical companies were associated with favourable conclusions on e-cigarettes. However, we did not find that authors openly reported COI in all cases; sometimes, this information was extracted based on the author affiliation, funding received in previous studies or from the acknowledgments section. We observed that some authors reported no COI but declared financial support from or even were employees of PTEC_CO (code 3 in our classification); thus, they did not acknowledge this potential COI. Whether non-reporting of COI was consciously done or not is beyond the aims of this study. Lack of transparency at the time of submission on these issues is likely to lead to a perception by readers and editors that the research is tainted^25^, thus is must be avoided.

### Limitations and strengths

Our analysis was limited to the information reported in the papers; thus, any financial ties or other interests that were not reported in the paper could not be assessed, so the COI may be underestimated. Also, not all journals specify whether all their publications are peer-reviewed; not conducting peer review may contribute to a lack of declaration of potential COI. In addition, we only searched publications in the PubMed database. However, PubMed is the core database for the biomedical sciences. Another limitation is the cross-sectional nature of this study, so finding causal relationships was beyond the scope of the study. In the search strategy, we included publications up to August 2014; this time-frame represents seven years after e-cigarettes launched into the market (2007 to 2014). Nevertheless, given the growing number of papers on e-cigarettes, it is possible that more recent publications would show different tendencies. However, we were interested in the situation when e-cigarettes first began to gain popularity, so the study period covered those years. Since some of the authors of this report have conducted or are conducting research on e-cigarettes, their judgments could also have some bias. However, all publications were assessed by pairs of reviewers who achieved a consensus; also, a protocol and algorithm were created to facilitate the objective assessment of each publication, and the procedures were pilot-tested. In addition, we only identified COI when authors reported them in any of the sections of the papers (including COI, acknowledgments and financial sections, as well as the affiliation information). This way, we were able to establish their ties with pharmaceutical, e-cigarette and tobacco companies. Future bibliographic and social science studies should be done to explore the several sources of COI in e-cigarette publications, besides what authors have reported in papers. Moreover, we only studied potential COI related to the authors, but COI related to the editors and the reviewers might also be relevant^26^. This type of study is difficult to design and conduct, since peer review is usually blinded and any potential COI are thus unclear. This raises the question of whether current journal policies are sufficient to ensure objectivity in the publication process and whether the current COI definitions and restrictions on publication should be expanded.

The study also has some strengths. It pioneered new methodology to describe COI according to the criteria proposed by the COPE^7^. In addition, it classified COI disclosures into 5 categories and created an algorithm to facilitate the evaluation, thus increasing internal validity. The algorithm allowed us to detect COI that could be misleading or that were not well-described. This tool might be useful for identifying situations in which authors do not correctly report their COI. In addition, the results of this study represent an exhaustive review of potential COI for a highly relevant topic, taking into account several publication-related variables.

## CONCLUSIONS

A third of the assessed publications about e-cigarettes did not have any COI disclosure, and this proportion was even higher in certain kinds of publications, such as news, editorials and other types of articles. Publications with favourable conclusions about e-cigarettes were more likely to include a COI disclosure. Furthermore, favourable conclusions about e-cigarettes were more frequent in publications that had COI declarations related to pharmaceutical companies. Journal editors and reviewers should consider evaluating publications, including the funding sources, to determine whether the results and conclusions may be biased and to determine whether there are any relationships with PTEC_CO.
