# Supplementary material for: Conflicts of interest in research on electronic cigarettes
Source: Tob Induc Dis. 2018 Jun 1;16:28. doi: 10.18332/tid/90668 (PMC6659563; doi:10.18332/tid/90668)
Supplement: Supplementary file 1 [file TID-16-28-s1.pdf]

Supplementary Figure S1. Algorithm used to assess the disclosure status of the electronic cigarette publications

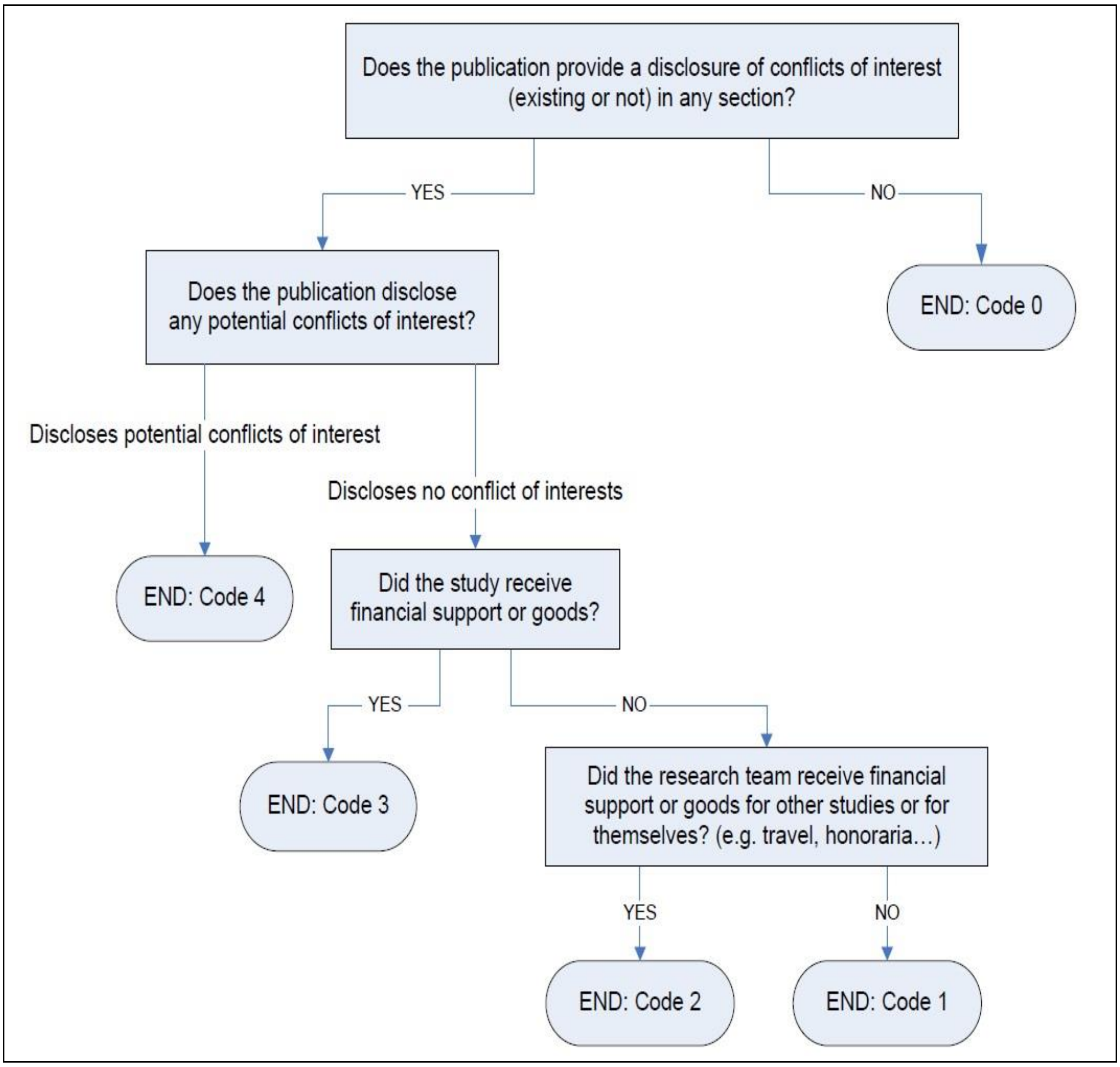

Code 0: The authors did not disclose any COI within the manuscript.

Code 1: The authors disclosed no COI, and no funding from PTEC_CO are mentioned.

Code 2: The authors disclosed no COI but acknowledged that some authors or previous studies they conducted received funding from PTEC_CO in the past.

Code 3: The authors disclosed no COI but acknowledged receiving funding from PTEC_CO.

Code 4: The authors disclosed COI with PTEC_CO.

PTEC_CO: pharmaceutical (P), tobacco (T) and/or electronic cigarette (EC) companies.

Supplementary Figure S2. Distribution of electronic cigarette publications according to their disclosure status by publication year.

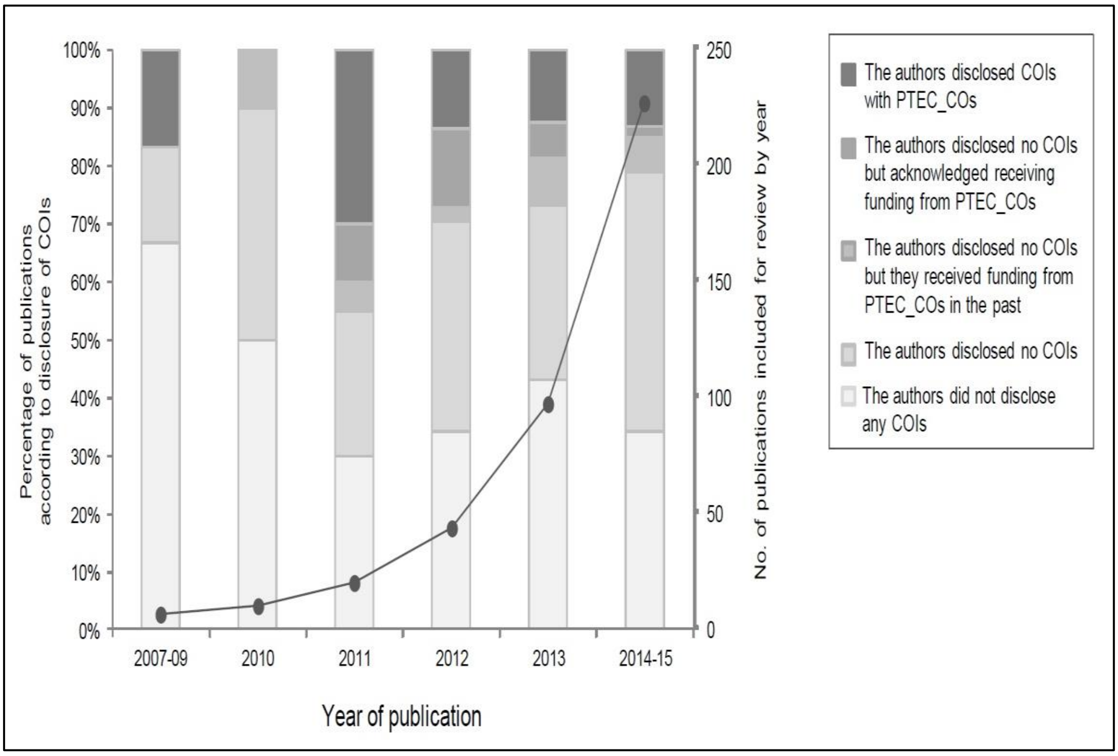

*Publications from 2014 (n=218) and 2015 (8 months; n=9) were combined.

COI: conflict of interest.
